# Correlates of poor oral health related quality of life in a cohort of people who use methamphetamine in Australia

**DOI:** 10.1186/s12903-023-03201-w

**Published:** 2023-07-13

**Authors:** Shady Abdelsalam, Michael Livingston, Brendan Quinn, Paul A Agius, Bernadette Ward, Lisa Jamieson, Paul Dietze

**Affiliations:** 1 National Drug Research Institute (NDRI), Faculty of Health Sciences, Curtin University, 85 Commercial Rd, Melbourne, VIC 3004 Australia; 2 Disease Elimination Program, Burnet Institute, 85 Commercial Rd, Melbourne, VIC 3004 Australia; 3 Centre for Alcohol Policy Research, La Trobe University, Bundoora, VIC 3086 Australia; 4 School of Public Health and Preventative Medicine, Monash University, 553 St Kilda Rd, Melbourne, VIC 3004 Australia; 5 Faculty of Health, Deakin University, Burwood, VIC 3125 Australia; 6 Monash University School of Rural Health, 26 Mercy St, Bendigo, VIC 3550 Australia; 7 Australian Research Centre for Population Oral Health, University of Adelaide Dental School, Adelaide, SA 5005 Australia

**Keywords:** Access to care, Cohort studies, Dental public health, Methamphetamine, Oral health related quality of life, Social determinants

## Abstract

**Objectives:**

Methamphetamine use impacts oral health, but little is known about its impacts on oral health related quality of life (OHRQoL). In this study we examined OHRQoL in a cohort of people who use methamphetamine and assessed associations with sociodemographic, behavioural, psychosocial and dental service utilisation correlates. A secondary aim was to examine the relationship between methamphetamine route of administration and OHRQoL, to test whether smoking the drug is associated with reduced OHRQoL.

**Methods:**

Cross-sectional analysis was performed, using data from VMAX, a cohort of people who use methamphetamine at least monthly in Victoria, Australia (n = 194). Utilising the oral health impact profile (OHIP-14), we assessed three OHRQoL outcomes: OHIP-14 prevalence, OHIP-14 extent and OHIP-14 severity. Regression analyses examined associations between independent variables and the three OHIP-14 outcome measures.

**Results:**

A significant segment of the cohort (35%) reported poor OHRQoL. Overall, no statistically significant association was detected between methamphetamine route of administration and the three OHIP-14 outcomes. Participants living in rural areas, with moderate-to-severe self-reported depression and with methamphetamine dependence had significantly worse OHRQoL levels, which persisted after adjusting for other covariates.

**Conclusion:**

Overall, VMAX cohort participants reported reduced OHRQoL levels. Our findings highlight the need for upstream interventions to improve the OHRQoL of people who use methamphetamine, with specific focus on those living in rural locations. Further research on the links between OHRQoL and mental health among people who use methamphetamine is required.

**Supplementary Information:**

The online version contains supplementary material available at 10.1186/s12903-023-03201-w.

## Introduction

Methamphetamine is a central nervous system stimulant, with an estimated 0.7% of all adults aged 15–64 having used the drug in 2020 [1]. These rates vary globally, with a reported use prevalence of 3.9% in North America and 1.3% in Australia and New Zealand in 2020 [1]. In addition to its desired effects, including euphoria and elevated self-confidence, methamphetamine use is associated with a range of short- and long-term negative effects such as cardiovascular problems, depression and psychotic symptoms [2]. Methamphetamine is typically injected, smoked or snorted, with injection providing the quickest onset of drug effects, closely followed by smoking [3, 4]. These two routes of administration result in an increased propensity for dependence and markedly worse health outcomes compared to other routes [5].

In Australia, the drug is typically found in three different forms: powder ‘speed’, crystalline ‘ice’ and paste ‘base’, with the crystalline form dominating the market since an upswing in availability in 2009 [6]. Crystalline methamphetamine is most frequently smoked rather than inhaled or injected in contemporary Australia [7]. The economic burden of methamphetamine in Australia in 2013/14 was estimated to have been approximately $5 billion [8]. These costs span the domains of premature mortality and morbidity, crime and those associated with service responses [8].

Methamphetamine use has been associated with bruxism, non-carious tooth wear and xerostomia [9], with some studies subsequently reporting increased rates of dental decay and oral disorders [10]. Studies utilising propensity score analyses have reported people who use methamphetamine having higher rates of untreated dental disease [11] and more missing teeth than comparable samples from the general population [5]. A reported two-fold increase in the prevalence of decayed, missing or filled teeth within a cohort of Americans who use methamphetamine, compared to the general population, further consolidates the association between methamphetamine use and oral disease [11]. In extreme cases, the detrimental oral health impacts of methamphetamine use result in a clinical presentation referred to as ‘meth mouth’, manifesting as rampant dental caries involving the facial and interproximal surfaces of maxillary anterior teeth [12].

Methamphetamine use has been shown to have a dose-response relationship with dental disease, with frequent drug use directly proportional to the incidence of oral disease [13]. However, the association between route of methamphetamine use and its effect on oral health is unclear. Smoking methamphetamine was originally hypothesised to have been responsible for increased rates of dental decay, the drug’s acidic nature having a caustic effect on tooth structure once inhaled, subsequently leading to meth mouth [14]. Conversely, cross-sectional studies have found a higher prevalence of dental decay, missing teeth and cosmetic problems amongst people who primarily inject methamphetamine, compared to those who smoke or snort the drug [11, 15]. Further aggravating their poor oral health status, people who use methamphetamine often have highly cariogenic diets, rich in carbonated drinks and sweets [16], and inadequate oral hygiene measures, compared to the general population [17]. Some authors have subsequently hypothesised that suboptimal oral health behaviours, entailing diet, oral hygiene and infrequent dental visits are the primary drivers behind poor oral health in people who use methamphetamine, rather than any aspect of methamphetamine use in itself [11, 15].

Poor oral health can have a significant impact on quality of life [18, 19]. Oral health related quality of life (OHRQoL) is a multidimensional concept, incorporating physical, psychological and social impacts of oral health [18, 20] and utilised to assess the overall effect of oral health on quality of life [19]. Instruments utilised to assess OHRQoL include the oral health impact profile (OHIP-14) [21], a validated instrument previously used to assess OHRQoL among samples of people who use drugs [22, 23]. Previous studies assessing OHRQoL in people who inject drugs revealed lower levels of oral health, compared to the general population [22–24].

Truong et al. found that the primary drug of choice (heroin vs. methamphetamine) does not appear to significantly alter OHRQoL scores [22], which suggests that the relationship between drugs injected and OHRQoL involved factors related to injecting drug use more broadly. Mukherjee and colleagues assessed OHRQoL in people who use methamphetamine, using a shortened version of the OHIP-14, and found a positive correlation between frequent methamphetamine use and non-smoking route of administration (such as injecting and inhalation) with some components of OHRQoL [24]. However, they did not present summary scores for OHIP measures, meaning that more work is needed to understand OHRQoL in all of the domains measured by the OHIP-14 in samples of people who use methamphetamine. Within cohorts of people who use drugs, sociodemographic correlates of poor OHRQoL included being female, older individuals, persons with lower levels of education and unemployed individuals [22–24]. Additionally, a significant positive association between depression and poor OHRQoL was reported by the authors in a cohort of Australian people who inject drugs [23].

## Aims

The primary aim of this exploratory study was to examine OHRQoL in a cohort of people who use methamphetamine and to examine associations with sociodemographic, behavioural, psychosocial and dental service utilisation correlates. We aim to achieve this by establishing a baseline record of OHRQoL in the VMAX cohort and comparing it to OHRQoL levels in select populations of both people who use drugs [22, 23] and the general population [25]. Given concerns around the impacts of methamphetamine smoking on oral health, a secondary aim of this study was to examine the relationship between methamphetamine route of use and OHRQoL, to test whether smoking the drug is associated with reduced OHRQoL.

## Methods

### Design and setting

We conducted a cross-sectional analysis of a sub-sample of the VMAX study; a prospective cohort of people who use methamphetamine. The methods have been described in detail elsewhere [26]. In summary, since 2016, participants have been recruited via a mix of respondent-driven sampling, convenience and targeted approaches, from metropolitan and rural areas of the state of Victoria, Australia [26]. VMAX interviews were conducted face-to-face at baseline, with subsequent face-to-face or telephone follow-up interviews conducted at up to six-month intervals [26]. Eligibility criteria included being 18 years of age or older and at least monthly use of methamphetamine via non-injecting routes in the preceding six months. Eligibility criteria regarding route of administration were relaxed when recruitment slowed; this resulted in 12% of the baseline participants reporting injecting as their preferred route of administration [26]. To measure OHRQoL, the OHIP-14 was introduced to the questionnaire in December 2019. From N = 853 VMAX participants with baseline interviews, n = 194 had at least one OHRQoL record. We selected the interview with the baseline OHRQoL record for each participant. Participants provided informed consent prior to undertaking the VMAX questionnaire. The VMAX study protocol was approved by the Alfred Hospital and Monash University Ethics Committees. This study is reported according to STROBE guidelines [27].

### Outcome measures

The OHIP-14 consists of 14 items used to measure seven self-reported domains related to oral health [21]. The tool has been widely used to measure OHRQoL in cohort studies [28] and population level studies [18]. Answers are recorded on a five-point Likert scale from 0 to 4, corresponding to 0 “*Never*”, 1 “*Hardly ever*”, 2 “*Occasionally*”, 3 “*Fairly often*” and 4 “*Very often*” [18]. Higher OHIP-14 scores are generally indicative of worse oral health. Replicating earlier studies [22, 23], we used three different OHIP-14 measures of OHRQoL:

#### OHIP-14 prevalence

A binary (yes/no) measure of the proportion of respondents who answered ‘fairly often’ or ‘very often’ to one or more of the OHIP-14 items. Participants in this study reporting OHIP-14 prevalence were considered to have worse OHRQoL, compared to their peers not reporting OHIP-14 prevalence.

#### OHIP-14 extent

A score of the number of items (0–14) for which respondents answered “fairly often” or “very often” [18]. Higher scores indicate worse OHRQoL.

#### OHIP-14 severity

The summary score (0–56) of participant’ responses to the OHIP-14 questions [18]. Higher scores indicate poorer OHRQoL.

OHRQoL, measured via the three OHIP-14 outcomes described above, will be presented for the VMAX cohort. Additionally, for comparative purposes, the corresponding OHIP-14 scores for the Melbourne Injecting Drug Use Cohort Study (SuperMIX) [23], the 2013 Illicit Drug Reporting System (IDRS) [22] and the 2004-06 National survey of adult oral health (NSAOH) conducted at the population level in Australia [25] will be presented.

### Exposure variables

A comprehensive set of variables measuring personal characteristics, drug use and dental service utilisation factors were included in our analyses, consistent with prior studies of similar cohorts [22, 23, 26]. To facilitate the interpretability and reproducibility of our findings, we dichotomised route of methamphetamine administration to indicate “Smoking” or “Other”, with “Other” defined as either oral ingestion, injecting or snorting of methamphetamine.

Sociodemographic factors included birthplace (Australia or Overseas), age (< 30 years, 30–40 years, > 40 years), sex (Male/Female), Aboriginal and Torres Strait Islander status (Yes/No), housing status (Stable/Unstable), with stable defined as home ownership, renting a home or residing at parents’ property. Unstable housing included boarding homes, couch surfing, homelessness and staying at an emergency home or shelter [29]. Other sociodemographic variables included recruitment location (metropolitan Melbourne/rural Victoria), highest level of education (< Year 10, Year 10–11, ≥Year12/Other) and employment status (Yes/No), with “Yes” encompassing both part-time and full-time employment.

Depression was determined using the patient health questionnaire (PHQ-9), a validated instrument used to assess depression progress and severity [30]. PHQ-9 scores range from 0 to 27, with higher scores indicating worse self-reported depression [30]. For the purposes of our research, and in line with other studies in the literature [30], a PHQ-9 cut-off score of 10 was utilised, with respondents’ scores categorised as “<10” or “≥10”, corresponding to “No-to-mild depressive symptoms” or “Moderate-to-severe depressive symptoms”, respectively.

Drug use variables included the participants’ drug of choice (methamphetamine, heroin, cannabis, other), current treatment for methamphetamine use (Yes/No), poly-drug use, defined as the use of methamphetamine and other illicit drugs in the previous month (Yes/No) and duration of methamphetamine use (< 10 years, 10–20 years, ≥ 20 years). Other drug-related variables included participants’ methamphetamine dependence, as indexed by the severity of dependence scale (SDS) [31]. SDS results were categorised as “<4” or “≥4”, with scores ≥ 4 suggestive of methamphetamine dependence [31]. Tobacco smoking in the previous month (Yes/No) was also included, in light of evidence showing that people who smoked both methamphetamine and cigarettes had a three-fold increase in dental decay, compared to their non-tobacco-smoking peers [13]. Data on dental service utilisation in the previous year were measured (public, private, none), corresponding to treatment at a public dental service, private practice or no dental visit, respectively.

### Statistical analyses

Descriptive statistics were generated for the exposure and outcome variables, with OHIP-14 outcomes contrasted against findings from comparable studies of people who use drugs and the general population. Four sequential regression models (Models A-D) were utilised for all three OHIP-14 outcomes: **Model A**: a bivariable model assessing the relationship between the route of methamphetamine administration and each OHIP-14 outcome; **Model B**: multivariable model adjusted for route of methamphetamine administration and personal characteristics; **Model C**: multivariable model adjusted for route of methamphetamine administration, personal characteristics and drug use variables; **Model D**: multivariable model adjusted for route of methamphetamine administration, personal characteristics, drug use variables and dental service variables. The fully adjusted models for all three OHIP-14 outcomes of interest are presented below, with the individual sequential regression analyses performed for each of the three OHIP-14 outcomes presented in the appendix to this study.

**Table 1:** Factors associated with OHIP-14 prevalence, extent and severity: Multivariate regression analyses showing adjusted risk ratio (ARR), 95% confidence interval (95% CI) (n = 194)

	OHIP-14 prevalence	OHIP-14 extent	OHIP-14 severity
**Variables**	**ARR** (95% CI)	**ARR** (95% CI)	**ARR** (95% CI)
**Most common route of methamphetamine use**			
Other	**Ref.**	**Ref.**	**Ref.**
Smoking	1.42 (0.91,2.21)	2.07 (0.94,4.56)	1.36 (0.81,2.28)
**Sex**			
Male	**Ref.**	**Ref.**	**Ref.**
Female	0.97 (0.65,1.44)	1.38 (0.64,2.99)	1.42 (0.81,2.48)
**Age group**			
< 30	**Ref.**	**Ref.**	**Ref.**
30–39	1.38 (0.73,2.64)	1.3 (0.4,4.29)	0.93 (0.41,2.12)
> 40	1.55 (0.72,3.34)	1.9 (0.44,8.09)	1.04 (0.38,2.83)
**Country of birth**			
Australia	**Ref.**	**Ref.**	**Ref.**
Other	0.78 (0.33,1.82)	0.57 (0.13,2.57)	0.78 (0.28,2.19)
**Aboriginal or Torres Strait Islander**			
No	**Ref.**	**Ref.**	**Ref.**
Yes	0.78 (0.41,1.49)	0.5 (0.14,1.76)	0.53 (0.23,1.27)
**Housing**			
Stable	**Ref.**	**Ref.**	**Ref.**
Unstable	0.83 (0.51,1.38)	1.14 (0.5,2.57)	1.41 (0.81,2.44)
**Recruitment location**			
Metropolitan Melbourne	**Ref.**	**Ref.**	**Ref.**
Regional Victoria	1.45 (0.92,2.28)	**3.14** (1.27,7.73) *	**2.2** (1.24,3.91) *
**Highest level of education**			
<Year 10	**Ref.**	**Ref.**	**Ref.**
Year 10–11	1.11 (0.64,1.91)	0.98 (0.36,2.69)	1.1 (0.57,2.11)
Year 12/Higher/Other	1 (0.53,1.88)	1 (0.37,2.72)	0.98 (0.48,2)
**Employment**			
No	**Ref.**	**Ref.**	**Ref.**
Yes	0.91 (0.54,1.53)	0.79 (0.35,1.82)	0.82 (0.46,1.45)
**Depression (PHQ-9 Score)**			
< 10 (No-mild symptoms)	**Ref.**	**Ref.**	**Ref.**
≥ 10 (Moderate-severe symptoms)	**1.91** (1.19,3.09) *	**3.95** (1.75,8.93) *	**2.29** (1.35,3.96) *
**Drug of choice**			
Methamphetamine	**Ref.**	**Ref.**	**Ref.**
Cannabis	1.41 (0.83,2.39)	**2.47** (1,6.13) *	1.67 (0.88,3.2)
Heroin	0.81 (0.23,2.78)	0.6 (0.09,3.41)	0.49 (0.15,1.65)
Other	1.14 (0.69,1.88)	1.19 (0.49,2.86)	1.06 (0.58,1.94)
**Poly-drug use**			
No	**Ref.**	**Ref.**	**Ref.**
Yes	0.82 (0.56,1.21)	0.93 (0.43,2.05)	0.92 (0.54,1.55)
**SDS score**			
< 4	**Ref.**	**Ref.**	**Ref.**
≥ 4 (suggestive of dependence)	1.53 (0.97,2.242)	1.79 (0.78,4.12)	**1.83** (1.04,3.21) *
**Current treatment for methamphetamine use**			
No	**Ref.**	**Ref.**	**Ref.**
Yes	1.29 (0.82,2.05)	1.03 (0.35,3.04)	1.03 (0.49,2.17)
**Duration of methamphetamine use**			
< 10 years	**Ref.**	**Ref.**	**Ref.**
10–20 years	1 (0.61,1.65)	1.31 (0.45,3.78)	1.4 (0.66,2.98)
≥ 20 years	1.03 (0.54,1.95)	1.15 (0.28,4.71)	1.57 (0.6,4.12)
**Dental service utilisation past year**			
None	**Ref.**	**Ref.**	**Ref.**
Public	1.03 (0.66,1.6)	0.71 (0.32,1.58)	0.94 (0.55,1.59)
Private	0.7 (0.31,1.56)	0.62 (0.21,1.83)	0.7 (0.34,1.44)

**OHIP-14 prevalence**: Reporting one or more OHIP item/s “fairly often” or “very often.”. Multivariate Poisson regression analysis was performed for the OHIP-14 Prevalence outcome, with results reported as adjusted risk ratio (ARR), 95% confidence interval (95% CI).

**OHIP-14 extent**: Number of items reported either “Very often” or “Fairly often”. Multivariate negative binomial regression models were utilised for the OHIP-14 Extent outcome, with results reported as adjusted risk ratio (ARR), 95% confidence interval (95% CI).

**OHIP-14 severity**: Sum of all ordinal responses. Multivariate negative binomial regression models were utilised for the OHIP-14 Severity outcome, with results reported as adjusted risk ratio (ARR), 95% confidence interval (95% CI).

*P-value ≤0.05

**SDS**: Severity of Dependence Scale.

Prior to conducting analyses, variance inflation factors (VIF) were estimated for covariates and possible factors contributing to model multicollinearity were identified. The variable “Smoked in the previous month” (VIF = 6.19) was subsequently dropped from our statistical analyses.

Given that OHIP-14 prevalence was a common outcome (> 10% prevalence), Poisson regression modelling with robust standard errors was used to provide unbiased estimates of risk. Odds ratios from logistic regression can overestimate risk when outcomes are common and Poisson regression on binary data with appropriate standard error estimation is less prone to bias under these conditions [32, 33]. In terms of analyses of OHIP-14 extent and severity data negative binomial regression modelling was used to provide unbiased estimates of risk in the presence of response over-dispersion [34]. Results from all bivariate regression models were reported as risk ratios (RRs) and adjusted risk ratios (ARRs) for all adjusted models, with 95% confidence intervals (95% CI) and probability values (p-values). A complete case approach to missing data was used for all analyses. Statistical significance for this study was set at p < 0.05 and Stata/SE 17 (Statacorp LLC, College Station, Texas, USA) was used for all statistical analyses.

## Results

### Participant characteristics

The characteristics of the VMAX sub-sample (n = 194) included in this study are described in Table 1. Most participants were from rural areas (64%) and a large minority (40%) reported PHQ-9 scores ≥ 10, suggesting moderate-to-severe depression. Over a third of the sample reported methamphetamine as their drug of choice, with almost two thirds having used it for over 10 years. Smoking methamphetamine was the route of administration of choice for most participants (60%). A large minority (43%) had SDS scores ≥ 4, suggestive of methamphetamine dependence, and about one in 10 reported being on treatment for methamphetamine use when interviewed. Nearly half (43%) the participants reported poly-drug use. Over half (54%) the study cohort had not seen a dentist in the previous year. Among participants who had visited the dentist, twice as many had used public rather than private dental services.

**Table 2:** Selected baseline summary characteristics (n = 194), December 2019-August 2020

	n (%)
**Sex**	
Male	114 (59)
Female	80 (41)
**Age**	
Mean age (SD)	37.3 (10)
< 30	46 (24)
30–39	82 (42)
> 40	66 (34)
**Ethnicity**	
Born in Australia	183 (94)
Born overseas	11 (6)
**Aboriginal and Torres Strait Islander**	
No	178 (92)
Yes	16 (8)
**Housing**	
Stable	141 (73)
Unstable	53 (27)
**Recruitment location**	
Metropolitan Melbourne	69 (36)
Rural Victoria	125 (64)
**Highest level of education**	
<Year 10	46 (24)
Year 10–11 Year 12/Higher/Other	85 (44) 63 (32)
**Employment**	
No	146 (75)
Yes	48 (25)
**Current treatment for methamphetamine use**	
No	171 (88)
Yes	23 (12)
**Drug of choice**	
Methamphetamine Cannabis	74 (36) 40 (20)
Heroin Other	10 (5) 80 (39)
**Poly-drug use in the last month** No (Methamphetamine only)	111 (57)
Yes (Poly-drug use)	83 (43)
**Severity dependence scale (SDS) score** < 4 ≥ 4 (Suggestive of methamphetamine dependence)	110 (57) 84 (43)
**Most common route of methamphetamine use**	
Other	78 (40)
Smoking **Duration of methamphetamine use** < 10 years 10–20 years ≥ 20 years **Smoked tobacco in the previous month** No	116 (60) 72 (37) 70 (36) 52 (27) 33 (17)
Yes	161 (83)
**Depression (PHQ-9 Score)**	
< 10 (No-mild depressive symptoms)	116 (60)
≥ 10 (Moderate-severe depressive symptoms)	78 (40)
**Dental service utilisation past year**	
None	104 (54)
Public Private	60 (31) 30 (15)
**OHIP Prevalence**	
Reporting one or more OHIP item/s “fairly often” or “very often”	67 (35)
**OHIP Extent** Number of OHIP items reported “fairly often” or “very often”	
Mean	1.58 (95%CI:1.15,2.01)
Median (25%,75% percentile)	0 (0,2)
**OHIP Severity**	
Mean of summary scores, ranging from 0–56	9.63 (95%CI:7.8,11.47)
Median score (25%,75% percentile)	3 (0,16)

A third of participants (n = 67, 35%) reported one or more OHIP-14 item ‘fairly often’ or ‘very often’ (prevalence). The OHIP-14 extent score was 1.58 (95%CI:1.15,2.01), while OHIP-14 severity was 9.63 (95%CI:7.8,11.47). Table 2 illustrates these results, comparing them with other cohorts of people who use drugs [22, 23] and the general population [25].

**Table 3:** OHIP-14 summary scores for selected cohorts

	VMAX (n = 194)	Melbourne Injecting Drug User Cohort Study (SuperMIX) (n = 943)†	2013 Illicit Drug Reporting System (IDRS) (n = 794)*	2004-06 National survey of adult oral health (NSAOH) (n = 3295)**
OHIP-14 Prevalence (95%CI)	**35%** (27.9,41.7)	**46%** (43.12,49.58)	**48%** (45,52)	**18.6%** (16.6,20.7)
Mean OHIP-14 Extent score (95%CI)	**1.58** (1.15,2.01)	**2.35** (2.12,2.57)	**2.5** (2.2,2.7)	**0.52** (0.44,0.59)
Mean OHIP-14 Severity score (95%CI)	**9.63** (7.8,11.47)	**12.72** (11.82,13.62)	**13.5** (12.5, 14.5)	**7.6** (7.1,8.1)

†Abdelsalam, Van Den Boom [23]

* Truong, Higgs [22]

**Crocombe, Mahoney [25]

### Factors associated with OHIP-14 prevalence, extent and severity

The fully adjusted multivariate regression models for the three OHIP-14 outcomes of interest are presented in Table 3. The fully adjusted sequential Poisson regression model for OHIP-14 prevalence exhibited a significant association between self-reported depression with reporting adverse oral health impacts. Individuals with PHQ-9 scores ≥ 10 had a higher risk of reporting OHIP-14 prevalence and worse OHRQoL, compared to participants with lower PHQ-9 scores.

The fully adjusted model for OHIP-14 extent, the outcome measure assessing the number of adverse oral health impacts reported by participants, is presented in Table 3. Participants recruited in rural Victoria were more likely to report higher OHIP-14 extent scores, compared to those recruited in metropolitan Melbourne. Participants categorised as experiencing moderate-to-severe depression were also more likely to report higher OHIP-14 extent scores than those who did not. Finally, participants reporting cannabis as their drug of choice had higher OHIP-14 extent scores than those reporting a preference for other drugs.

Multivariable analysis for the OHIP-14 severity outcome is shown in Table 3, highlighting a significant association between both recruitment location and moderate-to-severe depression with higher OHIP-14 severity scores. These findings displayed a similar pattern to those observed for OHIP-14 extent. Respondents screened as methamphetamine dependent, with SDS scores ≥ 4, were also more likely to report higher OHIP-14 severity scores in the fully adjusted analysis, compared to those with SDS scores < 4.

Overall, no statistically significant association was estimated between route of methamphetamine administration and the three OHIP-14 outcomes.

## Discussion

Our exploratory study showed that our sample of participants from the VMAX cohort reported worse OHRQoL compared to the general Australian population [25], but higher OHRQoL compared to other studies of people who use drugs in Australia [22, 23]. Data from comparable studies of populations of people who use drugs and the general population are presented for comparison purposes in Table 2. This finding was consistent across all three measures of OHRQoL. Screening positive for depression amongst methamphetamine users was a risk factor for poor OHRQoL across all measures. While there was evidence of poorer OHRQoL among participants who screened positive for methamphetamine dependence or who lived in rural locations, this pattern was not statistically significant across all measures. There was no association between route of methamphetamine administration and poor OHRQoL. Previous research has indicated that factors such as high sugar consumption, xerostomia and lack of oral hygiene behaviours contributed more to adverse oral health impacts than route of administration [15].

We found very few demographic factors were related to poor OHRQoL of participants. One exception was residing in a rural location, which was generally associated with poorer OHRQoL than residing in metropolitan Melbourne. These findings may reflect a lack of available services in rural localities, as reflected by lower dental attendance rates and worse levels of general and oral health amongst individuals residing in non-metropolitan Australia overall [35]. This has subsequently led to people residing in regional and remote areas being designated as a ‘priority population’ in Australia’s National Oral Health Plan 2015–2024, with the aim of improving oral health outcomes and dental service accessibility for this cohort [36]. The intersectionality of barriers to obtaining care, which include drug dependence, lack of accessible and adequate treatment services, poor mental health, and geographic remoteness [37], suggest that providing dental healthcare services via a more holistic, upstream approach linked to mental health and drug dependence treatment could have OHRQoL benefits.

Moderate-to-severe depression, as indexed by the PHQ-9, was frequently reported by cohort members, a finding noted in other studies of people who use methamphetamine [38]. We found consistent associations between depression and OHRQoL measures, similar to those found in a study of people who inject drugs in Melbourne [23]. Mukherjee et al. reported worse OHRQoL in people who use methamphetamine who had also reported depression or anxiety [24]. The direction of the relationship between depression and OHRQoL is not yet clear but, as indicated, it does highlight potential OHRQoL impacts of addressing mental health issues among people who use methamphetamine and the insight that longitudinal analysis of this relationship may provide.

Another potential ramification of VMAX participants having a higher prevalence of depression, compared to the general population [39], was lower dental service utilisation. Less than half of the VMAX cohort had seen a dentist in the previous year, similar to findings reported within other Australian cohorts of people who use methamphetamine [40]. This compares unfavourably with the 56.4% dental attendance rate of the Australian adult population in 2017-18 [35]. Our findings are consistent with studies highlighting the detrimental effect of depression on both dental service utilisation [41] and dental health status [42], suggesting a possible link between depression and poor oral health outcomes in the VMAX cohort. Furthermore, VMAX respondents who had visited a dentist were twice as likely to visit a public dental service, where they often encountered longer waiting times to access treatment and are frequently offered problem-oriented treatment options, instead of more complex restorative and preventive treatment modalities [43].

Participants reporting cannabis as their preferred drug had worse OHIP-14 extent scores, consistent with findings of worse oral health outcomes amongst people who use cannabis chronically in the literature [10]. Similarly, a cohort study of individuals with substance use disorders in Norway reported an association between frequent cannabis use with poor OHRQoL levels [44]. We present this association with interest, noting the documented association between frequent cannabis use and depression [45] and given that 41% of the VMAX study sample had moderate-to-severe self-reported depression.

We found methamphetamine dependence to be associated with poorer OHRQoL, in two of the three OHIP-14 outcome measures assessed. Despite 42% of the cohort having SDS scores suggestive of methamphetamine dependence, only 11% were enrolled in treatment for methamphetamine use at the time of interview. A potentially relevant contributing factor to reduced treatment service uptake is ‘service avoidance’, which occurs when people who use methamphetamine consider themselves sufficiently functional within society and do not perceive the need for professional service utilisation [46]. Quinn et al. found that ‘service avoiding’ people who use methamphetamine were more likely to predominantly smoke the drug, compared to more ‘service inclined’ people [46].

The absence of an association between route of methamphetamine use and OHRQoL within the VMAX cohort highlights the need for more integrated, upstream interventions to ameliorate oral health outcomes for people who use methamphetamine via any route of administration. Harm reduction and preventive interventions should be designed to address the social determinants of oral health inequalities [47], which may subsequently improve OHRQoL outcomes and overcome structural barriers to adequate dental care for people who use methamphetamine. This will allow for the provision of comprehensive dental care in a systematic manner, which has been shown to result in improved OHRQoL in other populations [25]. In turn, improved OHRQoL results in better overall quality of life and general wellbeing [20]. Given how poor oral health potentially contributes to depression [48], improved OHRQoL in people who use methamphetamine may also have a positive impact on mental health outcomes and overall wellbeing. Finally, there is need for more treatment interventions for people who use methamphetamine, tailored to meet the demographically diverse needs of people from different types of locations and within different age brackets [49].

### Limitations

Compared to other studies assessing OHRQoL in Table 2, our sample size was relatively smaller. The use of self-reported measures in the data collection of this study raises the possibility of recall and social desirability bias. Some of the instruments used, such as PHQ-9, have not been validated in cohorts of people who use methamphetamine. However, the validity and accuracy of self-reported data collected from drug use [50] and oral health [51] surveys has been documented in the literature. The variable “Smoked in the previous month” was identified as possibly contributing to model multicollinearity and was subsequently dropped; however, prior to dropping the variable from our analyses, we did not detect a statistically significant association between the variable and any of the three OHRQoL outcomes. This finding comes despite established links between smoking and poor OHRQoL reported in other cohort [52] and population studies [53]. Other contributors to poor oral health outcomes and ultimately affecting OHRQoL, such as xerostomia [9], were not assessed. Finally, the cross-sectional framework utilised in this study precludes inferring causality to observed associations. Longitudinal data with this cohort will be used to disentangle the complex relationship between depression, methamphetamine dependence and OHRQoL and consider the potentially confounding effects of structural barriers to care and poly-drug use, including cannabis.

## Electronic supplementary material

Below is the link to the electronic supplementary material.

Supplementary Material 1

## Data Availability

The data that support the findings of this study are available on request from the corresponding author. The data are not publicly available due to privacy or ethical restrictions.
