# Supplementary material for: Correlates of poor oral health related quality of life in a cohort of people who use methamphetamine in Australia
Source: BMC Oral Health. 2023 Jul 13;23:479. doi: 10.1186/s12903-023-03201-w (PMC10339651; doi:10.1186/s12903-023-03201-w)
Supplement: Supplementary file 1 — Supplementary Material 1 [file 12903_2023_3201_MOESM1_ESM.docx]

**Supplementary File**

| **Table S1. Factors associated with OHIP-14 Prevalence:** Sequential Poisson regression analyses showing risk ratio (RR), adjusted risk ratio (ARR), 95% confidence interval (95% CI) ……… (n=194) | | | | | | | | |
| --- | --- | --- | --- | --- | --- | --- | --- | --- |
| **Model A** | | **Model B** | | **Model C** | | **Model D** | |  |
| **Variables** | **RR** (95% CI) | **P-value** | **ARR** (95% CI) | **P-value** | **ARR** (95% CI) | **P-value** | **ARR** (95% CI) | **P-value** |
| **Most common route of methamphetamine use** |  |  |  |  |  |  |  |  |
| Other | **Ref.** |  | **Ref.** |  | **Ref.** |  | **Ref.** |  |
| Smoking | 1.2 (0.8,1.81) | 0.37 | 1.46 (0.96,2.23) | 0.08 | 1.39 (0.89,2.18) | 0.14 | 1.42 (0.91,2.21) | 0.13 |
| **Sex** |  |  |  |  |  |  |  |  |
| Male |  |  | **Ref.** |  | **Ref.** |  | **Ref.** |  |
| Female |  |  | 0.95 (0.65,1.39) | 0.81 | 0.95 (0.64,1.4) | 0.8 | 0.97 (0.65,1.44) | 0.87 |
| **Age group** |  |  |  |  |  |  |  |  |
| <30 |  |  | **Ref.** |  | **Ref.** |  | **Ref.** |  |
| 30-39 |  |  | 1.43 (0.81,2.52) | 0.22 | 1.37 (0.72,2.62) | 0.34 | 1.38 (0.73,2.64) | 0.32 |
| >40 |  |  | 1.53 (0.82,2.86) | 0.18 | 1.56 (0.72,3.36) | 0.26 | 1.55 (0.72,3.34) | 0.27 |
| **Country of birth** |  |  |  |  |  |  |  |  |
| Australia |  |  | **Ref.** |  | **Ref.** |  | **Ref.** |  |
| Other |  |  | 0.82 (0.36,1.88) | 0.64 | 0.75 (0.32,1.75) | 0.5 | 0.78 (0.33,1.82) | 0.56 |
| **Aboriginal or Torres Strait Islander** |  |  |  |  |  |  |  |  |
| No |  |  | **Ref.** |  | **Ref.** |  | **Ref.** |  |
| Yes |  |  | 0.89 (0.49,1.62) | 0.70 | 0.8 (0.42,1.51) | 0.49 | 0.78 (0.41,1.49) | 0.45 |
| **Housing** |  |  |  |  |  |  |  |  |
| Stable |  |  | **Ref.** |  | **Ref.** |  | **Ref.** |  |
| Unstable |  |  | 0.84 (0.53,1.33) | 0.46 | 0.86 (0.54,1.36) | 0.51 | 0.83 (0.51,1.38) | 0.48 |
| **Recruitment location** |  |  |  |  |  |  |  |  |
| Metropolitan Melbourne |  |  | **Ref.** |  | **Ref.** |  | **Ref.** |  |
| Regional Victoria |  |  | 1.55 (0.99,2.42) | **0.05*** | 1.45 (0.91,2.3) | 0.12 | 1.45 (0.92,2.28) | 0.11 |
| **Highest level of education** |  |  |  |  |  |  |  |  |
| <Year 10 |  |  | **Ref.** |  | **Ref.** |  | **Ref.** |  |
| Year 10-11 |  |  | 1.07 (0.64,1.8) | 0.79 | 1.15 (0.67,1.98) | 0.61 | 1.11 (0.64,1.91) | 0.71 |
| Year 12/Higher/Other |  |  | 0.92 (0.5,1.69) | 0.78 | 0.97 (0.51,1.85) | 0.94 | 1 (0.53,1.88) | 1 |
| **Employment** |  |  |  |  |  |  |  |  |
| No |  |  | **Ref.** |  | **Ref.** |  | **Ref.** |  |
| Yes |  |  | 0.92 (0.55,1.52) | 0.74 | 0.84 (0.51,1.38) | 0.49 | 0.91 (0.54,1.53) | 0.72 |
| **Depression (PHQ-9 Score)** |  |  |  |  |  |  |  |  |
| <10 (No-mild symptoms) |  |  | **Ref.** |  | **Ref.** |  | **Ref.** |  |
| ≥10 (Moderate-severe symptoms) |  |  | 2.34 (1.53,3.58) | **<0.001*** | 1.94 (1.2,3.13) | **0.01** | 1.91 (1.19,3.09) | **0.01*** |
| **Drug of choice** |  |  |  |  |  |  |  |  |
| Methamphetamine |  |  |  |  | **Ref.** |  | **Ref.** |  |
| Cannabis |  |  |  |  | 1.42 (0.83,2.42) | 0.2 | 1.41 (0.83,2.39) | 0.2 |
| Heroin |  |  |  |  | 0.82 (0.24,2.81) | 0.75 | 0.81 (0.23,2.78) | 0.73 |
| Other |  |  |  |  | 1.12 (0.68,1.85) | 0.65 | 1.14 (0.69,1.88) | 0.61 |
| **Poly-drug use** |  |  |  |  |  |  |  |  |
| No |  |  |  |  | **Ref.** |  | **Ref.** |  |
| Yes |  |  |  |  | 0.82 (0.55,1.21) | 0.32 | 0.82 (0.56,1.21) | 0.33 |
| **SDS score** |  |  |  |  |  |  |  |  |
| <4 |  |  |  |  | **Ref.** |  | **Ref.** |  |
| ≥4 (suggestive of dependence) |  |  |  |  | 1.54 (0.98,2.42) | 0.06 | 1.53 (0.97,2.42) | 0.07 |
| **Current treatment for methamphetamine use** |  |  |  |  |  |  |  |  |
| No |  |  |  |  | **Ref.** |  | **Ref.** |  |
| Yes |  |  |  |  | 1.34 (0.84,2.13) | 0.22 | 1.29 (0.82,2.05) | 0.27 |
| **Duration of methamphetamine use** |  |  |  |  |  |  |  |  |
| <10 years |  |  |  |  | **Ref.** |  | **Ref.** |  |
| 10-20 years |  |  |  |  | 1.01 (0.61,1.66) | 0.98 | 1 (0.61,1.65) | 0.99 |
| ≥20 years |  |  |  |  | 1.02 (0.54,1.95) | 0.94 | 1.03 (0.54,1.95) | 0.93 |
| **Dental service utilisation past year** |  |  |  |  |  |  |  |  |
| None |  |  |  |  |  |  | **Ref.** |  |
| Public |  |  |  |  |  |  | 1.03 (0.66,1.6) | 0.91 |
| Private |  |  |  |  |  |  | 0.7 (0.31,1.56) | 0.38 |

**OHIP-14 Prevalence:** Reporting one or more OHIP item/s “fairly often” or “very often”**. Model A:** Key exposure (route of methamphetamine use) and OHIP-14 outcome; **Model B:** Adjusted for key exposure and personal characteristics; **Model C:** Adjusted for key exposure, personal characteristics and drug use variables**; Model D:** Adjusted for key exposure, personal characteristics, drug use variables and dental service variables. *P-value significance set at p≤0.05. **SDS**: Severity of Dependence Scale.

| **Table S2. Factors associated with OHIP-14 Extent:** Sequential negative binomial regression models showing risk ratio (RR), adjusted risk ratio (ARR), 95% confidence interval (95% CI) ……… (n=194) | | | | | | | | |
| --- | --- | --- | --- | --- | --- | --- | --- | --- |
|  | **Model A** | | **Model B** | | **Model C** | | **Model D** | |
| **Variables** | **RR** (95% CI) | **P-value** | **ARR** (95% CI) | **P-value** | **ARR** (95% CI) | **P-value** | **ARR** (95% CI) | **P-value** |
| **Most common route of methamphetamine use** |  |  |  |  |  |  |  |  |
| Other | **Ref.** |  | **Ref.** |  | **Ref.** |  | **Ref.** |  |
| Smoking | 0.98 (0.5,1.93) | 0.95 | 1.58 (0.75,3.33) | 0.23 | 1.9 (0.88,4.1) | 0.1 | 2.07 (0.94,4.56) | 0.07 |
| **Sex** |  |  |  |  |  |  |  |  |
| Male |  |  | **Ref.** |  | **Ref.** |  | **Ref.** |  |
| Female |  |  | 1.25 (0.58,2.67) | 0.57 | 1.4 (0.64,3.04) | 0.4 | 1.38 (0.64,2.99) | 0.42 |
| **Age group** |  |  |  |  |  |  |  |  |
| <30 |  |  | **Ref.** |  | **Ref.** |  | **Ref.** |  |
| 30-39 |  |  | 1.47 (0.61,3.54) | 0.39 | 1.32 (0.4,4.33) | 0.64 | 1.3 (0.4,4.29) | 0.66 |
| >40 |  |  | 2.17 (0.83,5.7) | 0.12 | 2.09 (0.49,8.85) | 0.32 | 1.9 (0.44,8.09) | 0.39 |
| **Country of birth** |  |  |  |  |  |  |  |  |
| Australia |  |  | **Ref.** |  | **Ref.** |  | **Ref.** |  |
| Other |  |  | 0.5 (0.12,2.17) | 0.36 | 0.53 (0.12,2.42) | 0.41 | 0.57 (0.13,2.57) | 0.46 |
| **Aboriginal or Torres Strait Islander** |  |  |  |  |  |  |  |  |
| No |  |  | **Ref.** |  | **Ref.** |  | **Ref.** |  |
| Yes |  |  | 0.44 (0.13,1.49) | 0.19 | 0.51 (0.14,1.78) | 0.29 | 0.5 (0.14,1.76) | 0.28 |
| **Housing** |  |  |  |  |  |  |  |  |
| Stable |  |  | **Ref.** |  | **Ref.** |  | **Ref.** |  |
| Unstable |  |  | 1.02 (0.49,2.11) | 0.97 | 0.98 (0.45,2.13) | 0.97 | 1.14 (0.5,2.57) | 0.76 |
| **Recruitment location** |  |  |  |  |  |  |  |  |
| Metropolitan Melbourne |  |  | **Ref.** |  | **Ref.** |  | **Ref.** |  |
| Rural Victoria |  |  | 3.49 (1.46,8.33) | **0.01*** | 3.26 (1.35,7.88) | **0.01*** | 3.14 (1.27,7.73) | **0.01*** |
| **Highest level of education** |  |  |  |  |  |  |  |  |
| <Year 10 |  |  | **Ref.** |  | **Ref.** |  | **Ref.** |  |
| Year 10-11 |  |  | 1.09 (0.46,2.58) | 0.85 | 0.97 (0.37,2.55) | 0.95 | 0.98 (0.36,2.69) | 0.97 |
| Year 12/Higher/Other |  |  | 1.18 (0.46,3.04) | 0.74 | 1.03 (0.38,2.81) | 0.95 | 1 (0.37,2.72) | 1 |
| **Employment** |  |  |  |  |  |  |  |  |
| No |  |  | **Ref.** |  | **Ref.** |  | **Ref.** |  |
| Yes |  |  | 0.93 (0.42,2.06) | 0.86 | 0.75 (0.33,1.68) | 0.48 | 0.79 (0.35,1.82) | 0.59 |
| **Depression (PHQ-9 Score)** |  |  |  |  |  |  |  |  |
| <10 (No-mild symptoms) |  |  | **Ref.** |  | **Ref.** |  | **Ref.** |  |
| ≥10 (Moderate-severe symptoms) |  |  | 5.03 (2.36,10.69) | **<0.001*** | 4 (1.77,9) | **0.001*** | 3.95 (1.75,8.93) | **0.001*** |
| **Drug of choice** |  |  |  |  |  |  |  |  |
| Methamphetamine |  |  |  |  | **Ref.** |  | **Ref.** |  |
| Cannabis |  |  |  |  | 2.54 (1.03,6.26) | **0.04*** | 2.47 (1,6.13) | **0.05*** |
| Heroin |  |  |  |  | 0.62 (0.1,3.78) | 0.6 | 0.6 (0.09,3.41) | 0.53 |
| Other |  |  |  |  | 1.16 (0.48,2.77) | 0.74 | 1.19 (0.49,2.86) | 0.7 |
| **Poly-drug use** |  |  |  |  |  |  |  |  |
| No |  |  |  |  | **Ref.** |  | **Ref.** |  |
| Yes |  |  |  |  | 1.01 (0.47,2.17) | 0.99 | 0.93 (0.43,2.05) | 0.87 |
| **SDS score** |  |  |  |  |  |  |  |  |
| <4 |  |  |  |  | **Ref.** |  | **Ref.** |  |
| ≥4 (suggestive of dependence) |  |  |  |  | 1.78 (0.78,4.05) | 0.11 | 1.79 (0.78,4.12) | 0.17 |
| **Current treatment for methamphetamine use** |  |  |  |  |  |  |  |  |
| No |  |  |  |  | **Ref.** |  | **Ref.** |  |
| Yes |  |  |  |  | 1.04 (0.36,3.06) | 0.94 | 1.03 (0.35,3.04) | 0.96 |
| **Duration of methamphetamine use** |  |  |  |  |  |  |  |  |
| <10 years |  |  |  |  | **Ref.** |  | **Ref.** |  |
| 10-20 years |  |  |  |  | 1.27 (0.44,3.68) | 0.65 | 1.31 (0.45,3.78) | 0.62 |
| ≥20 years |  |  |  |  | 1.03 (0.25,4.21) | 0.97 | 1.15 (0.28,4.71) | 0.85 |
| **Dental service utilisation past year** |  |  |  |  |  |  |  |  |
| None |  |  |  |  |  |  | **Ref.** |  |
| Public |  |  |  |  |  |  | 0.71 (0.32,1.58) | 0.4 |
| Private |  |  |  |  |  |  | 0.62 (0.21,1.83) | 0.39 |

**OHIP-14 Extent:** Number of items reported either “Very often” or “Fairly often”**. Model A:** Key exposure (route of methamphetamine use) and OHIP-14 outcome; **Model B:** Adjusted for key exposure and personal characteristics; **Model C:** Adjusted for key exposure, personal characteristics and drug use variables**; Model D:** Adjusted for key exposure, personal characteristics, drug use variables and dental service variables. *P-value significance set at p≤0.05. **SDS**: Severity of Dependence Scale.

| **Table S3. Factors associated with OHIP-14 Severity:** Sequential negative binomial regression models showing risk ratio (RR), adjusted risk ratio (ARR), 95% confidence interval (95% CI) ……… (n=194) | | | | | | | | |
| --- | --- | --- | --- | --- | --- | --- | --- | --- |
|  | **Model A** | | **Model B** | | **Model C** | | **Model D** | |
| **Variables** | **RR** (95% CI) | **P-value** | **ARR** (95% CI) | **P-value** | **ARR** (95% CI) | **P-value** | **ARR** (95% CI) | **P-value** |
| **Most common route of methamphetamine use** |  |  |  |  |  |  |  |  |
| Other | **Ref.** |  | **Ref.** |  | **Ref.** |  | **Ref.** |  |
| Smoking | 0.91 (0.56,1.48) | 0.7 | 1.14 (0.68,1.91) | 0.61 | 1.31 (0.78,2.19) | 0.3 | 1.36 (0.81,2.28) | 0.25 |
| **Sex** |  |  |  |  |  |  |  |  |
| Male |  |  | **Ref.** |  | **Ref.** |  | **Ref.** |  |
| Female |  |  | 1.18 (0.69,2.02) | 0.54 | 1.39 (0.8,2.43) | 0.25 | 1.42 (0.81,2.48) | 0.22 |
| **Age group** |  |  |  |  |  |  |  |  |
| <30 |  |  | **Ref.** |  | **Ref.** |  | **Ref.** |  |
| 30-39 |  |  | 1.17 (0.63,2.15) | 0.62 | 0.93 (0.41,2.12) | 0.86 | 0.93 (0.41,2.12) | 0.87 |
| >40 |  |  | 1.36 (0.7,2.71) | 0.39 | 1.07 (0.39,2.92) | 0.89 | 1.04 (0.38,2.83) | 0.94 |
| **Country of birth** |  |  |  |  |  |  |  |  |
| Australia |  |  | **Ref.** |  | **Ref.** |  | **Ref.** |  |
| Other |  |  | 0.95 (0.34,2.65) | 0.93 | 0.74 (0.26,2.09) | 0.5 | 0.78 (0.28,2.19) | 0.63 |
| **Aboriginal or Torres Strait Islander** |  |  |  |  |  |  |  |  |
| No |  |  | **Ref.** |  | **Ref.** |  | **Ref.** |  |
| Yes |  |  | 0.61 (0.25,1.45) | 0.26 | 0.54 (0.22,1.28) | 0.16 | 0.53 (0.23,1.27) | 0.15 |
| **Housing** |  |  |  |  |  |  |  |  |
| Stable |  |  | **Ref.** |  | **Ref.** |  | **Ref.** |  |
| Unstable |  |  | 1.32 (0.78,2.22) | 0.3 | 1.36 (0.79,2.34) | 0.27 | 1.41 (0.81,2.44) | 0.23 |
| **Recruitment location** |  |  |  |  |  |  |  |  |
| Metropolitan Melbourne |  |  | **Ref.** |  | **Ref.** |  | **Ref.** |  |
| Rural Victoria |  |  | 1.99 (1.14,3.48) | **0.02*** | 2.22 (1.26,3.93) | **0.01*** | 2.2 (1.24,3.91) | **0.01*** |
| **Highest level of education** |  |  |  |  |  |  |  |  |
| <Year 10 |  |  | **Ref.** |  | **Ref.** |  | **Ref.** |  |
| Year 10-11 |  |  | 1.09 (0.59,2.01) | 0.79 | 1.14 (0.6,2.17) | 0.68 | 1.1 (0.57,2.11) | 0.78 |
| Year 12/Higher/Other |  |  | 0.91 (0.46,1.81) | 0.78 | 0.98 (0.48,1.99) | 0.96 | 0.98 (0.48,2) | 0.96 |
| **Employment** |  |  |  |  |  |  |  |  |
| No |  |  | **Ref.** |  | **Ref.** |  | **Ref.** |  |
| Yes |  |  | 0.94 (0.53,1.64) | 0.83 | 0.77 (0.44,1.36) | 0.37 | 0.82 (0.46,1.45) | 0.49 |
| **Depression (PHQ-9 Score)** |  |  |  |  |  |  |  |  |
| <10 (No-mild symptoms) |  |  | **Ref.** |  | **Ref.** |  | **Ref.** |  |
| ≥10 (Moderate-severe symptoms) |  |  | 2.87 (1.73,4.74) | **<0.001*** | 2.31 (1.36,3.93) | **0.002*** | 2.29 (1.35,3.96) | **0.002*** |
| **Drug of choice** |  |  |  |  |  |  |  |  |
| Methamphetamine |  |  |  |  | **Ref.** |  | **Ref.** |  |
| Cannabis |  |  |  |  | 1.72 (0.9,3.28) | 0.1 | 1.67 (0.88,3.2) | 0.12 |
| Heroin |  |  |  |  | 0.51 (0.15,1.72) | 0.28 | 0.49 (0.15,1.65) | 0.25 |
| Other |  |  |  |  | 1.04 (0.57,1.9) | 0.9 | 1.06 (0.58,1.94) | 0.86 |
| **Poly-drug use** |  |  |  |  |  |  |  |  |
| No |  |  |  |  | **Ref.** |  | **Ref.** |  |
| Yes |  |  |  |  | 0.93 (0.55,1.56) | 0.78 | 0.92 (0.54,1.55) | 0.74 |
| **SDS score** |  |  |  |  |  |  |  |  |
| <4 |  |  |  |  | **Ref.** |  | **Ref.** |  |
| ≥4 (suggestive of dependence) |  |  |  |  | 1.85 (1.06,3.24) | **0.03*** | 1.83 (1.04,3.21) | **0.04*** |
| **Current treatment for methamphetamine use** |  |  |  |  |  |  |  |  |
| No |  |  |  |  | **Ref.** |  | **Ref.** |  |
| Yes |  |  |  |  | 1.06 (0.51,2.22) | 0.88 | 1.03 (0.49,2.17) | 0.94 |
| **Duration of methamphetamine use** |  |  |  |  |  |  |  |  |
| <10 years |  |  |  |  | **Ref.** |  | **Ref.** |  |
| 10-20 years |  |  |  |  | 1.45 (0.68,3.08) | 0.33 | 1.4 (0.66,2.98) | 0.38 |
| ≥20 years |  |  |  |  | 1.55 (0.59,4.07) | 0.37 | 1.57 (0.6,4.12) | 0.36 |
| **Dental service utilisation past year** |  |  |  |  |  |  |  |  |
| None |  |  |  |  |  |  | **Ref.** |  |
| Public |  |  |  |  |  |  | 0.94 (0.55,1.59) | 0.81 |
| Private |  |  |  |  |  |  | 0.7 (0.34,1.44) | 0.34 |

**OHIP-14 Severity:** Sum of all ordinal responses**. Model A:** Key exposure (route of methamphetamine use) and OHIP-14 outcome; **Model B:** Adjusted for key exposure and personal characteristics; **Model C:** Adjusted for key exposure, personal characteristics and drug use variables**; Model D:** Adjusted for key exposure, personal characteristics, drug use variables and dental service variables. *P-value significance set at p≤0.05. **SDS**: Severity of Dependence Scale.
